# Investigation of the Functional Ageing of Conductive Coated Fabrics under Simulated Washing Conditions

**DOI:** 10.3390/ma16030912

**Published:** 2023-01-18

**Authors:** Christian Biermaier, Phillip Petz, Thomas Bechtold, Tung Pham

**Affiliations:** 1 Research Institute of Textile Chemistry and Textile Physics, Universität Innsbruck, Hoechsterstrasse 73, 6850 Dornbirn, Austria; 2 Embedded Systems Laboratory, University of Applied Sciences Upper Austria, 4232 Hagenberg, Austria

**Keywords:** conductive textiles, metal–polymer adhesion, functional wash ageing, electroless copper deposition

## Abstract

Conductive textiles play an important role in recent electronics development; however, one of the major challenges remains their machine-washing durability. For the investigation of the basic wash ageing mechanisms, we used copper-plated polyamide 66 and cellulose fabrics and developed a wet and dry operable flex tester with online resistance recording. The evaluation was supported by abrasion tests, cyclic elongation tests and tribological investigation of dry and wet textile–textile friction. It was found that the contribution of mechanical and chemical ageing to wash ageing strongly depends on the substrate material. A bad adhesion of copper on polyamide 66 leads to early fatigue while better stability of the copper on cellulose leads to a stronger resistance against ageing. For both substrates, the delamination of the copper layer was the root cause of the fatigue, which is facilitated by the washing solution. Finally, a cumulative fatigue model was developed and the determination of the end of lifetime by the intended use is discussed.

## 1. Introduction

Electronic textiles are a growing segment of the electronics market [1] but also attract wide scientific interest. The field is interdisciplinary and comprises all stages from basic chemical [2] and material [3] developments to electrical engineering [4], textile design [5] and application [6]. The basis of e-textiles is the integration of conductive materials into non-conductive flexible textile substrates. The metallisation of polymers, e.g., by electroless copper deposition, reaches a high integration [2,7,8,9,10,11]. For real-life applications, the challenge of these hybrid materials is to achieve maximum durability against external impacts and treatments. Accelerated ageing through cyclic long-term durability testing of conductive textiles often leads to functional ageing due to material failure [12]. Bending, stretching and abrasion cause mechanical ageing. Aqueous solutions and oxygen cause chemical ageing. Wash ageing consists of certain aspects of mechanical treatment and chemical impact. It is also one of the most important real-life challenges and of high significance. Soft washing methods such as laboratory beaker washing, only have a low-stress intensity [13,14,15]. Exemplarily, the standard EN ISO 6330 for machine washing was often applied to e-textiles for durability testing [16,17,18]. It includes several washing parameters among which are detergent concentration, temperature, speed, cycle duration, loading mass, etc. However, it was conceived for conventional textile colour fastness testing with a domestic washing machine and lacks comparability because of a broad variety of washing programmes as discussed by Rotzler et al. [19] pointing out the need for standardisation of e-textile testing.

Nevertheless, the standard EN ISO 6330 was often used as a basis for the investigation into the mechanisms of functional wash ageing of metallic silver coated polyamide 66 yarns. Ismar et al. investigated their chemical ageing. The metallised yarns were exposed to water or detergent solution in concentration and temperature regarding EN ISO 6330 but without motion. Silver ablation was found as material damage and caused a small increase in electrical resistance [20]. Additives, such as oxidants, accelerated chemical ageing [21]. The mechanical part of wash ageing was investigated by Zaman et al. for embroidered metallised yarns [22]. The specimens underwent standard abrasion and pilling tests. Intermediate electrical resistance measurement showed a loss of conductivity with the increase in the cycle number. Additionally, they performed EN ISO 6330 conform cyclic washing. The authors used mathematical linear fittings on the course of the electrical resistance to find a possible substitution of wash cycles by abrasion cycles. However, the modelling only worked for selected data sets. The assumption that abrasion has the highest contribution to wash ageing was derived from work on the conventional textile washing efficiency by textile–textiles friction of dirty clothing by Lee et al. [23]. Atakan et al. combined the effects of water and mechanical treatment by performing wet abrasion tests on the silver metal plated polyamide yarns. The water strongly accelerated functional ageing compared to dry-state ageing [24]. Similar was found by Zaman et al. [25]. Two examples of protection against wash ageing apply lamination with thermoplastic polyurethane or protective embroidery on top of the conductive line. Both approaches slowed down the abrasive impact [26].

Those studies showed that machine washing of conductive textiles is a complex process with different interacting types of stress. A major issue is the contribution of the chemical and mechanical part wash ageing. From this, the question arises, if the ageing compartments show cumulative damage or at least cumulative gain of the electrical resistance [12,27,28]. For deeper investigation of the washing behaviour of metallised fabrics, the online resistance recording was considered a helpful tool, as shown in our previous work [10]. In this work, we developed a wet and dry operable dynamic flex tester, according to the principles described by de Kok et al. [29] that includes bending, stretching and abrasive components. Furthermore, we investigated the impact of water on the textile–textile friction for the estimation of the contribution of abrasion to wash testing. 

## 2. Materials and Methods

### 2.1. Materials

Trisodium citrate dihydrate (C_6_H_5_Na_3_O_7_·2H_2_O, p.a.) was purchased from Merck KGaA, Darmstadt, Germany. Silver nitrate (AgNO_3_, 99.9% p.a.), copper sulphate pentahydrate (CuSO_4_·5H_2_O, ≥99.5%), potassium hydrogen-L-tartrate (C_4_H_5_KO_6_, ≥99.0%), formaldehyde (H_2_CO, ≥ 37.0% in water/methanol) were supplied by Carl ROTH GmbH & Co. KG, Germany and sodium hydroxide (NaOH, ≥50% solution) was obtained from Deuring GmbH & Co. KG, Gemeinde Hörbranz, Austria. Two different fabrics are used in the study. The plain woven Lyocell fabric was kindly provided by Lenzing AG, Austria (127 g/m^2^, 39 warp/cm, 30 weft/cm, staple yarn, laser-cut: 2.5 × 22 cm^2^) and used as received. The plain woven PA 66 fabric was kindly provided by Getzner GmbH, Austria (83 g/m^2^, 40 warp/cm, 33 weft/cm, filament yarn, laser-cut: 2.5 × 22 cm^2^). The choice of Lyocell and PA 66 is made because the materials are the best representative examples. While cellulosic fibre (Lyocell) is one of the most widely used regenerated bio-based materials in textiles, PA 66 fibre is widely used in technical textiles and functional garments such as outdoor clothing. Furthermore, the two fibres are different in chemical structure and physical properties. Lyocell is highly hydrophilic due to the abundance of hydroxyl groups and thus exhibits much higher moisture sorption capacity compared to the more hydrophobic PA 66. 

Washing: the PA 66 specimens were washed for 30 min with a ratio of 10 specimens per 200 mL deionised water with 1.0 g Genapol LRO (fatty alcohol ether sulphate sodium salt, 25 w%) and 1.0 g Na_2_CO_3_ at 60 °C. The specimens were subsequently washed 4 times with 300 mL deionised water at 60 °C and once with 300 mL water at 20 °C. The specimens were dried for 5 min at 120 °C in an air ventilation oven. The electrical resistance of the pristine specimens was >10 MΩ·cm^−1^.

### 2.2. Tribological Investigation

**Figure 1:**
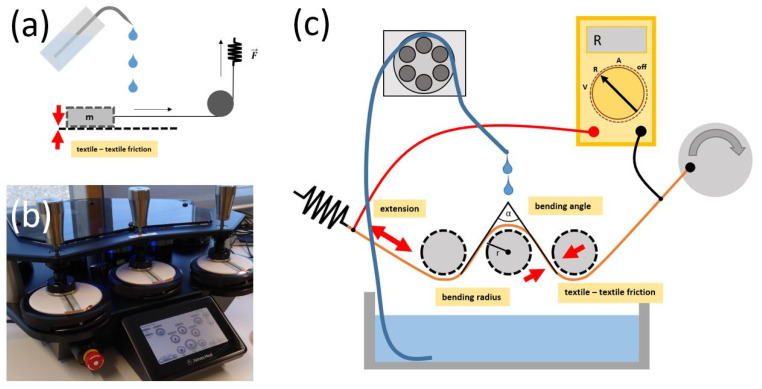
Experimental setups: (**a**) force measurement of the textile–textile friction, (**b**) treatment with a Martindale abrasion tester and (**c**) sketch of the newly developed dynamic flex tester in the setup in full washing mode with a conductive specimen.

A dry and pristine fabric of 8 × 12 cm^2^ was attached to a wooden rectangle plate of 5.2 × 10.1 cm^2^. The plate with the fabric was loaded with an iron weight. The full load (iron + wood) was measured as 12.4 N. The loaded rectangle was pulled over a second fabric of 13 × 30 cm^2^ for a distance of 8 cm with a tensile test device (Allroundline extensometer with 1 kN force cell, Zwick-Röll, Ulm, Germany) and the aid of a pulley and a carbon fibre multifilament. A sketch of the setup is depicted in Figure 1a. The force was measured for the fabric combinations Lyocell-Lyocell, PA 66-PA 66 and Lyocell-PA 66 in a dry state and in a wet state, i.e., with overflowing water on a plane table. For evaluation, only the force of the constant dynamic friction was used. 

### 2.3. Conductive Coating of the Fabrics

The manufacturing methods and conditions have been previously described elsewhere [2,10]. The Lyocell and PA 66 specimens were impregnated with a 0.19 M (5 w%) trisodium citrate solution dihydrate by Foulard treatment at 1 m/min and 3 bar pressurised air (Mathis HVF 33593, Werner Mathis AG, Oberhasli, Switzerland) and subsequently dried at 120 °C for 7 min at 750 rpm in an air ventilation oven. The PA 66 specimens were additionally loaded with 0.19 M trisodium citrate solution with an airbrush pen with good lab judgement and dried again. The additional citrate helped to decrease the spreading of the ink deposit. An additive-free aqueous 0.29 M (4.7 w%) silver nitrate ink was deposited by a valve jet printer model with a fixed nozzle head (J. Zimmer GmbH, Kufstein, Austria) with a mass flow of 0.3 g/min. The specimen was moved under the nozzle with a conveyor belt (GFV2G50S, Oriental Motor CO) at 3 m/min for the Lyocell specimens and 7 m/min for the PA 66 specimens to create a straight silver-containing pre-catalyst line. After thermal treatment at 160 °C and 2300 rpm for 20 min in an air ventilation oven (Mathis Labdryer LTE, Werner Mathis AG, Oberhasli, Switzerland), electroless copper deposition (ECD) was performed for 4 h at 20 °C (thermostat) in 90 mL of an aqueous 28 mM CuSO_4_, 240 mM NaOH, 78 mM KH-tatrate and 350 mM formaldehyde solution. Compared to a former publication [2], no sodium carbonate was used. The copper content as a gain of weight was found to be 32% for PA 66 and 21% for Lyocell based on the weight of the fabric below the conductive line.

### 2.4. Cyclic Abrasion

Abrasion was performed with a Martindale tester (James Heal, Halifax, UK). The specimens were fixed in the bottom circle mount (Figure 1b). Standard worsted wool was attached to the top stamp. The abradant was loaded with the 12 kPa labelled weight and moved back and forth with a speed of 47.5 rpm. The electrical resistance was recorded online. The Lyocell specimens were abraded 10,000 cycles on the top side and the PA 66 specimens 100,000 cycles on each side.

### 2.5. Cyclic Extension

The cyclic shoe sole bending tester BPM 3054 (PFI, Pirmasens, Germany) was reconstructed for a cyclic extension by removing a bending hinge. The specimens were extended at a frequency of 53 cycles per minute with an online resistance recording. The PA 66 specimen was extended to 0.40% of the initial length for 950,000 cycles. The Lyocell specimen was extended to 0.34% of the initial length for 503,000 cycles and additionally to 0.37% until summed up at 1,000,000 cycles.

### 2.6. Laser Scanning Microscopy (LSM)

The damages of the conductive line were pictured with a confocal 3D laser scanning microscope (VK-X150, Keyence corporation, Osaka, Japan) with a magnification of 20 fold.

### 2.7. Dynamic Flex Tester

A variant of a dynamic flex tester (following de Kok et al. [29]) with three rolls (Figure 1c and Supporting information—Figure S1) was developed. The dimension parameters of the device are listed in Table 1. The first and the second roll treated the front and the backside of a mounted specimen (Supporting information—Figure S2) and the third roll returned the tail of the specimen to the initial level for fixation. A sawn Lyocell fabric hose was wrapped around each roll for achieving textile–textile friction. The rolls were fixed on a steel spindle, which was fit into ball bearings for free rotation. The ball bearings were mounted to a support, which was attached to an aluminium profile (40 × 40) framework. Two rotatable disks with a steel spindle worked as a crank. Two metal springs in serial connection opposed the crank for pulling and straining the specimens in the opposite direction. The rolls, disks and support were 3D-printed of ABS. The crank was driven by a 72 W IKA lab motor. The specimens were inserted between the rolls and mounted to two polymer tent clamps that were attached to either the crank or the springs. The tent clamps provided the possibility of mounting a litze wire cable as well. Hence, the electrical contact was established by pressing copper metal wires onto copper metal deposits within the pressure points of the clamps.

The apparatus was operated in a dry state for simulating the pure mechanical contribution to wash ageing. For the simulation of full washing, a pot with 1 L washing solution was placed under the rolls. A peristaltic pump conveyed the liquid for dripping onto the middle roll with approximately 30 mL/min. By application of the solution without mechanical motion of the specimen, the chemical ageing during washing was simulated. The washing solution was composed of soft water with 5 g Genapol LRO 25% and 5 g Na_2_CO_3_ per litre. All washing experiments were conducted at room temperature. The electrical resistance was recorded with a U1733C LCR-meter (Keysight Technologies Deutschland GmbH, Böblingen, Germany).

## 3. Results and Discussion

### 3.1. Dry and Wet Textile–Textile Friction

For a better estimation of the contribution of abrasion to wash ageing, a tribological investigation was performed. Pristine fabrics were used in the setup shown in Figure 1a regarding Formula (1) in simplified friction theory.

(1) $$F_{f}=\mu *F_{n}$$

**Figure 2:**
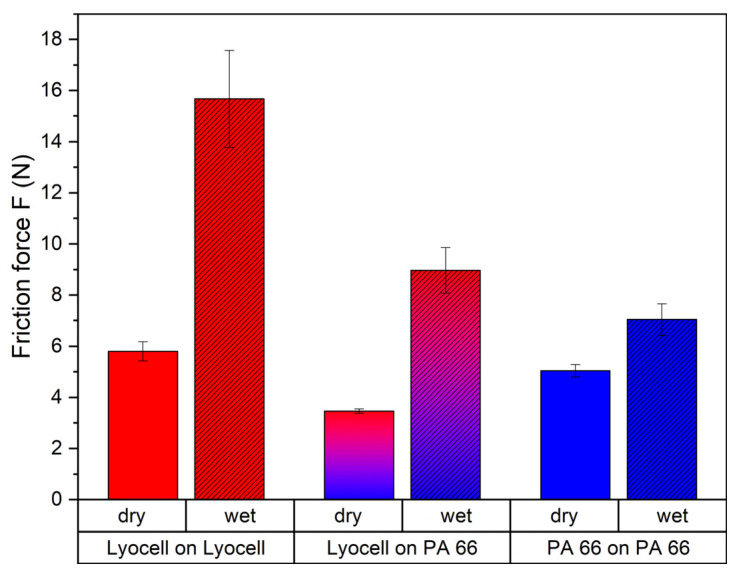
Wet and dry dynamic friction forces between Lyocell and polyamide 66 fabrics with an increase from dry to wet of 2.7-fold for Lyocell on Lyocell, 2.6-fold for Lyocell on PA 66 and 1.4-fold for PA 66 on PA 66.

The friction force *F_f_* is dependent on the normal force *F_n_*, which was kept constant in all experiments. The friction coefficient *µ* is dependent on the material combination. Hence, a comparison of the friction forces shows a comparison of the intensity of the friction. Figure 2 shows the measured forces for different fabric combinations. The differences between dry and wet friction forces are from interest. The friction force between two wet Lyocell fabrics was about 2.7 fold higher than between two dry Lyocell fabrics. A similar value was determined for the friction between a polyamide and a Lyocell fabric with a 2.6-fold force. For two polyamide fabrics, a multiplication of 1.4-fold from dry to wet friction force was found. These observations can be attributed to two effects. At first, the shearing force of the water was increased due to the capillary effect between the two surfaces [30]. Secondly, an assumedly larger effect, especially for Lyocell, may be the swelling of the fibres by soaking water and which led to an increased surface area [31]. The higher friction of wet fabrics suggests that the presence of water intensified the mechanical impact of washing compared to the dry state. 

### 3.2. Cyclic Extension and Abrasion Testing

**Figure 3:**
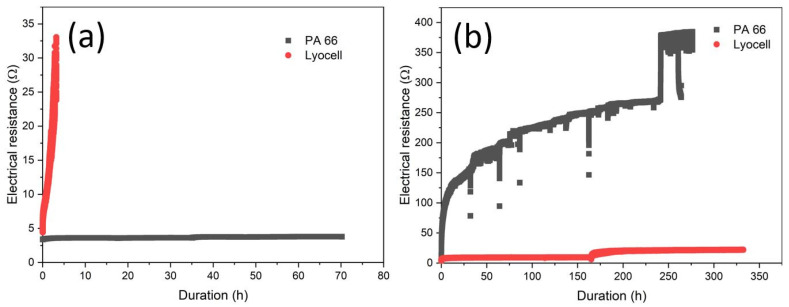
The course of the electrical resistance during mechanical ageing treatment at (**a**) cyclic abrasion with a Martindale tester and (**b**) cyclic stretching.

**Figure 4:**
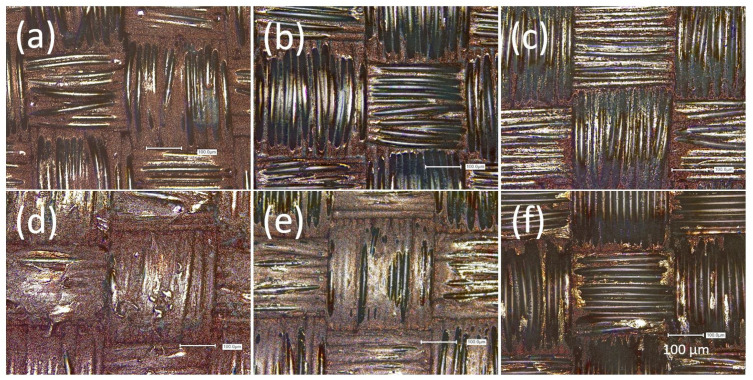
LSM images of copper-plated PA 66 after (**a**) manufacturing, (**b**) abrasion testing, (**c**) cyclic stretching, (**d**) dry flex testing, (**e**) chemical ageing and (**f**) full washing with a magnification of 20-fold.

**Figure 5:**
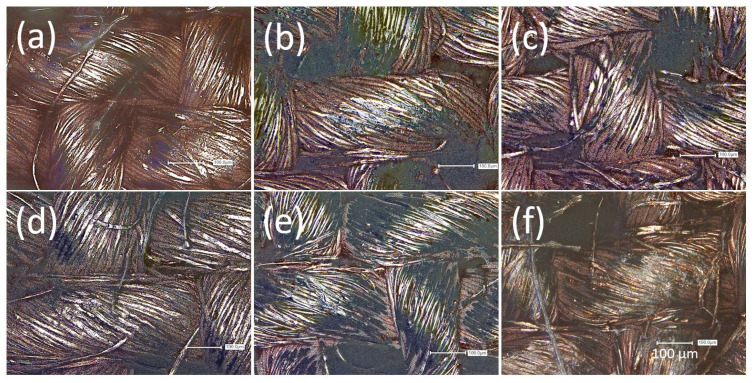
LSM images of copper-plated Lyocell after (**a**) manufacturing, (**b**) abrasion testing, (**c**) cyclic stretching, (**d**) dry flex testing, (**e**) chemical ageing and (**f**) full washing with a magnification of 20-fold.

The conductive specimens underwent mechanical durability testing, i.e., abrasion testing using the Martindale tester and cyclic extension using the modified shoe sole bending tester, for damage comparison in later wash testing. The resulting electrical resistance curves are shown in Figure 3. The PA 66 specimen did not show any significant increase in the electrical resistance over 100,000 abrasion cycles on each side (Figure 3a). The LSM imaging (Figure 4b) revealed that the bump-like exposed areas of the fabric showed delamination of the copper metal. In the slots at the yarn crossings, the copper remained and ensured conductive percolation. Thus, not the metal–polymer adhesion but the surface morphology of the fabric acted as abrasion protection against functional fatigue, as the abradant was not able to permeate the fabric. The Lyocell specimen fatigued due to substrate failure during abrasion after 10,000 cycles (Supporting information—Figure S3). This was seemingly caused by degradation effects during the thermal treatment of the citrate-impregnated specimens after the printing step [32]. Thus, a lower temperature and shorter duration of the thermal treatment would enhance the abrasion stability of the Lyocell substrate. The damage to the copper layer was found as delamination of the metal from the polymer at the exposed areas of the yarns (Figure 5b).

For the cyclic extension of the PA 66 specimen, a strong increase in the electrical resistance at the beginning of the treatment, with a flattening at a high level was found (Figure 3b). The change in resistance after ca 250 h could be due to the delamination but also the re-agglomeration of copper particles during the stretching process. However, the trends of the increased resistance are retained. The damage evaluation via LSM depicted the strong crack formation and delamination of the copper layer (Figure 4c). The cyclic extension of the Lyocell specimen did not show fatigue of lifetime over 1 M cycles (Figure 3b). A small step in the course of the electrical resistance was found after increasing the extension rate. The damage evaluation (Figure 5c) showed yarn rearrangements and shifts, especially at the yarn crossings. The difference between the two substrates is due to the different fabric structures and experimental setups. The cyclic extension in the used setup is distance related and not force related. The structure of the Lyocell fabric allowed extension by yarn deformation, whereas the extension of the PA 66 fabric was rather attributed to filament stretching with a smaller contribution of yarn deformation (c.f. stress–strain behaviour of the fabrics, Supporting information—Figure S4). The different Young’s moduli of copper and PA 66 led to tension at the interfaces and caused the breakage of the copper layer and delamination.

### 3.3. Ageing under Simulated Conditions in the Dynamic Flex Tester

**Figure 6:**
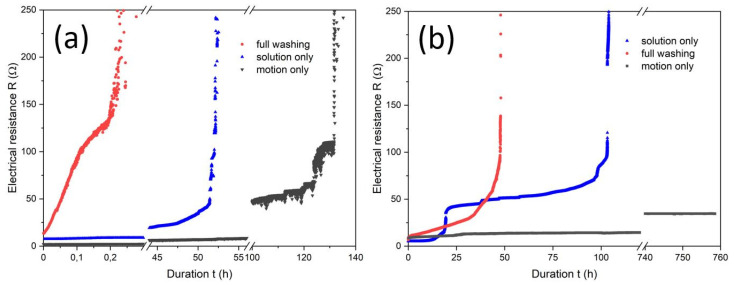
Course of the electrical resistance in the flex tester during dry dynamic flex testing, chemical ageing and full washing for (**a**) PA 66 specimens and (**b**) Lyocell specimens.

The course of the electrical resistance of the PA 66 specimen in the dynamic flex tester is shown in Figure 6a. All treatments show a less intensive increase in electrical resistance at the beginning. A loss of the conductive percolation can be considered at the nearly vertical increase in the electrical resistance. The “motion only” mode as dry dynamic flex testing simulated the mechanical part of the wash ageing. The relevant specimen failed after about 120 h, which is equal to 432,000 revelation cycles. The LSM imaging in Figure 4d showed cracking of the copper layer as the major damage. The cracking occurs especially at the yarn crossings (c.f. cyclic stretching). Thus, the damage is attributed to stretching and bending. The latter can be seen as a variant of stretching as demonstrated by Komolafe et al. by classical beam theory [12,33]. Furthermore some delamination was found. 

For the simulation of the chemical ageing (“solution only”), the specimen was placed in the flex tester under a supply of washing solution but without revelation. The specimen fatigued after 50 h. The metallic copper was assumedly oxidised by oxygen from air [34,35], which was abundant due to the experimental setup. The oxidation of copper metal in alkaline media generally leads to a porous cuprous oxide film with further oxidation to copper(II), as described by Ives et al. [35]. The corrosion of copper metal is a first-order reaction and is dependent on the oxygen concentration in the solution [36]. Herein, for the conductive line it means that the higher the duration of the ageing, the less copper metal is available for conductivity. The removal of copper is shown in Figure 4e. We assume that water-insoluble copper oxides, hydroxides or carbonates (due to the presence of sodium carbonate) were dissolved due to complexation by the surfactants.

The wet dynamic flex test (“full washing”) showed a high slope of the initial increase in the electrical resistance and loss of conductive percolation after 10 min of washing. As the progress of the chemical ageing is dependent on the duration of solution treatment, within the short time of 10 min chemical ageing by dissolution did not play a role. Thus, the main contribution of wash ageing was dedicated to mechanical treatment. However, the increase in the friction force of a factor of 2.6 by water, as demonstrated in the tribology measurements above, was far too low, for explaining the early fatigue. The LSM imaging proved that the failure was due to copper metal delamination from the polyamide fabric (Figure 4f). The patterns of the delamination showed a strong similarity to the abrasion-tested samples (Figure 4b), as mainly the exposed parts of the fabrics were delaminated and copper was still found in the deeper slots at the yarn crossings. From this we deduced, that the washing solution strongly lowered the adhesion at the metal–polyamide interface and the abrasion by textile–textile friction removed the copper layer under highly eased circumstances.

The Lyocell specimens were treated under the same conditions as the PA 66 specimens. The dry dynamic flex testing showed a slight increase in the electrical resistance (Figure 6b) and was aborted after cumulated one month of continuous operation (2.7 M revelation cycles) as no prompt failure was expected.

The LSM imaging in Figure 5d showed slight abrasion damage on the fabric’s surface (c.f. cyclic abrasion Figure 5b). The high stability of the copper on the Lyocell substrate may be due to swelling during the manufacturing and shrinking after drying, which contracts the pores/void volume and thus binds the copper mechanically [31,37,38].

A step in the course of the resistance for the chemical ageing of coated Lyocell was observed after 20 h leading to a higher resistance compared to the fully washed treated specimen. This change can be explained by the localised delamination and thus loss of some copper parts along the printed conductive line as a result of the inhomogeneity of the deposition and the adhesion of the coated layer to the fabric. However, the resistance remains at a lower level of about 50 Ω and the specimen can still be considered conductive. The total loss of the conductive percolation and thus, functional failure of the specimen was observed after approximately 90 h which is much longer compared to the failure caused by the full washing conditions (Figure 6b). This is nearly double the duration to failure as for PA 66, possibly caused by the different distribution of the copper in the fabric (Figure 4a and Figure 5a) and the different surficial concentrations of silver catalyst from manufacturing. As for PA 66, the failure is due to the removal of the copper layer by the dissolution of assumedly oxidised copper metal (Figure 5e).

The Lyocell specimen from the full washing lost its percolation after about 40 h. It is significantly higher than for PA 66 with 10 min. Obviously, the adhesion of the copper metal to the cellulose surface is strongly increased in comparison to PA 66. Lyocell has a high surface and a distinct pore structure, which could be a major contributing factor [31,39]. This includes the swelling/shrinking in wet/dry treatments [38] which may contribute to the physical binding of the metal. Another factor for the strong adhesion can be the high density of surficial hydroxyl groups [40]. However, cellulose surfaces at ambient conditions are covered with different intensively bound water layers among which are freezing bound water and non-freezing bound water [40,41,42,43,44]. It remains unclear if this kind of water is contributing to the metal–polymer adhesion mechanism, as there is also water to (copper) metal surface adsorption [45,46]. PA 66 in counter shows lower polarity and its wetting behaviour is dependent on the number of accessible polymer end-groups, i.e., amines and acids. Furthermore, the surfaces of the polymer appeared quite smooth [47]. 

The LSM imaging of the washing damages of the Lyocell specimen (Figure 5f) showed a loss of copper mainly in the exposed areas of the yarns. The comparison with the damage formation of abrasion testing (Figure 5b) indicated that abrasion played a major role in fatigue. Similar to PA 66 it can be assumed that the washing liquid lowered the metal–polymer adhesion and thus increased the effect of the abrasion. In counter to the PA 66 substrate, where chemical ageing did not play a role at full washing, the duration of 40 h until fatigue of Lyocell for full washing takes 44% of the duration of the chemical ageing until fatigue. Hence, the chemical contribution may also have accelerated the wash ageing. Despite the delamination of copper being found as a major issue for the washing of copper-plated Lyocell fabrics, the metal–polymer adhesion issue is not as crucial as for PA 66. A protective coating for the metal layer against the washing solution becomes more prominent within the topic of counteracting wash ageing. Another strategy for preventing or reducing delamination is to improve the interface adhesion between the conductive coating layer and textile substrate by surface activation of the fabrics such as, e.g., cryogenic treatment [48], plasma activation [49] or solubilisation activation [11]. 

### 3.4. Damage Modelling Theory

In our previous work [12], the cumulative fatigue damage modelling in the style of Palmgren and Miner was suggested for modelling the current damage as the sum of the partial damages in conductive textiles. The results from above, however, demonstrated that wash ageing is in general not simply the direct sum of the mechanical ageing and the chemical ageing. This is due to the different mechanisms that contribute to the damage formation. It can be expressed in (2) with *D* as the damage parameter.

(2) $$D_{wash}\neq \overset{m}{\underset{i=1}{\sum }}D_{mechanical}+\overset{m}{\underset{i=1}{\sum }}D_{chemical}$$

In order to combine chemical ageing and mechanical ageing in a summarising formula, we propose to use their time dependence. As mentioned above, chemical ageing is dependent on the duration of the washing solution treatment. The copper metal corrosion then can simplified be described by a corrosion rate *r_c_*. The mechanical impact is dependent on the number of flex cycles. This is related to the number of revelation cycles, which is time-dependent by the fixed revelation rate of 60 rpm of the lab motor. This results in a more descriptive formula for wash ageing (3).

(3) $$D_{wash}=a*b*D_{mechanical}+D_{chemical}=a*b*\frac{t_{i}}{t_{fmech}\left(I_{cyc},v_{cyc}\right)}+\frac{t_{i}}{t_{fchem}\left(r_{c}\right)}=\frac{t_{i}}{t_{fwash}}$$

Herein, *t_i_* is the time point of view and *t_fmech_* the duration to failure by mechanical ageing, which is dependent on cycle intensity *I_cyc_* and the cycle velocity *v_cyc_*. As the effect of mechanical ageing is strongly influenced by the presence of the washing liquid, factors *a* and *b* adjust the term of mechanical ageing to the washing reality. Factor *a* describes the increase in the textile–textile friction by water, i.e., the ratio between wet and dry friction forces (data given in Figure 2). The ratio of the friction force between wet and dry for the combination PA 66 and Lyocell (PA 66 on Lyocell in wrapped flex tester roll) was found as 2.6. Factor *b* describes the increased impact of the abrasion by weakening the metal–polymer adhesion. In our setup, *b* cannot be measured directly but can be calculated based on the value of factor *a*. For PA 66 substrate, the duration until failure because of mechanical treatment is 600 times higher than the duration until failure because of wash ageing, i.e., a∗b=600. As the chemical ageing by dissolution does not play a role in mechanical and wash ageing for PA 66, factor *b* is then calculated as 600:2.6 = 231. The partition of the mechanical damage into subcategories was neglected for simplification reasons. The duration of the chemical ageing until failure *t_fchem_* is dependent on the corrosion rate *r_c_*. Considering the chemical part of the wash ageing for PA 66, the comparison of the duration of wash ageing to failure and chemical ageing to failure results in a contribution of 0.4% to the potential damage by chemical ageing.

In the original Palmgren and Miner approach, an object is considered fatigued, when the damage parameter D reaches the value 1 (100% damage) by definition. The combination of such different kinds of ageing contributions as in this work made clear that there must be room for fatigue of the conductive percolation at damage parameters different from the value 1. An exception was if one damage parameter dominated strongly and was not affected by the other contributions. Hence, the damage values at failure comprise a distinct area of values.

### 3.5. Prediction of Ageing

For the characterisation of the material properties, the electrical resistance was used as an indicator of the conductive percolation within the copper-plated line on the fabric. A dramatic increase in the electrical resistance marked the end of lifetime. A finer differentiation can be made based on the intended application and connected devices as the conductive line may function as an energy supply or for data transmission. Based on hypothetical examples and demonstrators, it needs to be determined at what point the textile would be no longer usable.

The use of the conductive line as an energy supply requires low electrical resistance to limit the loss of energy. The power that is lost during transmission in the form of heat is proportional to the voltage across the conductor and the current flowing through it. The power loss in watts can be determined using the following formula:

(4) $$P_{Textile}\left(W\right)=R_{Textile}\left(\Omega \right)*I_{\mu C}{\left(A\right)}^{2}$$

With *P_Textile_* as electrical power that was lost, *R_Textile_* as electrical resistance of the conductive line to the device and back and *I_µC_* as the defined required current of a hypothetical microcontroller. The microcontroller is drawn as a fitting example, as it is commonly operated with a current requirement of, e.g., 40 mA, 80 mA and 160 mA at an input voltage of 5 V. The resistance of the conductive line leads to a certain voltage drop. Hence, the input voltage *U_Input_* must exceed the required 5 V at the device for the demanded constant current. This can be expressed by the formula:

(5) $$U_{Input}\left(V\right)=R_{Textile}\left(\Omega \right)*I_{\mu C}\left(A\right)+5V$$

In addition to the current demand for the microcontroller, the energy that can be provided by the power source is also relevant for estimating the fault condition in the textile. Not all battery-powered systems can compensate for the rise in resistance in the conductive textile and provide enough voltage. In Supporting information—Figure S5, three data points are highlighted at a textile resistance of 200 Ω, 500 Ω and 1000 Ω, which would require 21 V, 45 V and 85 V at a current of 40 mA. These voltages can no longer be provided by boost, flyback and forward converters from a 4.2 V lithium–polymer battery. The maximum possible resistance of the textile would therefore be limited to 160 Ω and a maximum input voltage of 18 V.

In contrast to power transmission, only a small current is required for digital data transmission. This is limited by the possible source and sink of the microcontroller pins and is assumed to be 20 mA. Due to simplicity, transmission is assumed to be UART transmission with one start bit and one stop bit. The rise time and fall time of a rectangular signal are independent of the voltage levels used as long as the required current can be provided and is given for a simple first-order RC network by:

(6) τ=R∗C

(7) $$t_{r}\tilde{=}\;2.2*\tau$$

With *C* as parasitic capacitance and *t_r_* as response time. For rule of thumb estimation, we assume *C* as 40 µF and *I* (include in R) as 20 mA. Hence, the Baud rate can be estimated in dependence on the resistance as shown in Table 2.

We deduce a maximum resistance of 2700 Ω for useful data transmission, which is higher than the 160 Ω for power transmission. These hypothetical estimations make clear, that the maximum tolerable resistance is setup dependent. It also underlines the need for correct calibration of integrated sensors, as the signals might be influenced by a drift of the electrical resistance. However, the above-shown steep increase in the electrical resistance during the loss of percolation at the end of the lifetime shows that the decision on a fixed resistance value for failure, such as 160 Ω or 2700 Ω, does not vary the lifetime by much due to the high slope.

**Figure 7:**
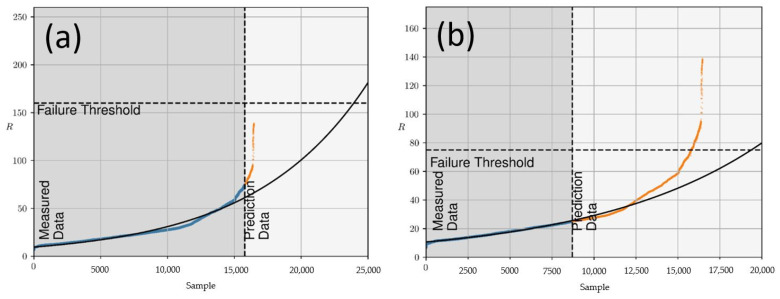
Polynomial prediction for full washing of the Lyocell specimen with (**a**) 75 Ω as prediction threshold and 160 Ω as failure threshold and (**b**) 25 Ω as prediction threshold and 75 Ω as failure threshold; the black line shows the polynomial function based on the prediction.

In a further step, the course of the resistance was predicted by fitting polynomial functions. A simulation threshold of 75 Ω was applied for gathering data. All values before reaching 75 Ω were used for calculating a further possible course of the resistance as shown in Figure 7a. Due to the sudden increase in the resistance and the high slope, the failure threshold of 160 Ω was reached with a strong discrepancy between the predicted and actual value. To lower the effect of the sudden fatigue, the failure threshold was adapted to those circumstances and set to 75 Ω, which is closer to the start of the high slope. However, this led to an adaption of the simulation threshold, too. We freely chose 25 Ω (Figure 7b). The new polynomial fit of the course of the electrical resistance in this iteration then met the failure threshold in a value closer to the real failure.

The prediction of the resistance behaviour added another parameter to the determination of the functional fatigue, besides the power transmission and the Baud rate. It moved the resistance where the conductive line is considered as fatigued to lower resistance values. This is because of the “nearly sudden fatigue” behaviour of the loss of percolation for this conductive line geometry and accelerated ageing setup. 

## 4. Conclusions

For the first time, an estimation of the contributions of chemical and mechanical ageing to wash ageing of metallised conductive textiles could be made by the comparison of the duration until failure in different operation modes of a newly developed wet flex tester. This was made possible by:

1. Using the electrical resistance as an indicator for function, i.e., conductive percolation.
2. The online resistance recording during treatment action.
3. The time dependence of the chemical ageing via the corrosion rate and the time dependence of the mechanical ageing via the motor drive of 60 rpm.
4. The separation of the ageing compartments, i.e., dry dynamic flex testing, wet dynamic flex testing and chemical ageing without motion.
5. The imaging of the surficial damages and comparison with conventional ageing treatment, e.g., classical abrasion testing.

The contribution of chemical ageing by the dissolution of the copper metal to the wash ageing of PA 66 fabrics was found very small. The major part was dedicated to mechanical ageing, as copper metal delamination was the reason for fatigue. In comparison to dry mechanical treatment, the delamination was strongly eased by the weakening of the adhesion of the metal to the polymer because of the presence of the washing solution. The magnitude of this effect exceeded an increase in the friction coefficient due to the presence of water by far. The Lyocell fabric outperformed the PA 66 fabric regarding washing durability far in excess because of the much higher metal–polymer adhesion. This led to a higher share of chemical ageing because of the longer-lasting influence of the washing solution. However, the weakening of the polymer–metal interface was still considered a major cause of the material ageing and fatigue. These findings demonstrate that PA 66 absolutely needed surface activation to enhance the metal–polymer adhesion for slowing down the delamination rate. As the Lyocell samples showed better adhesion the emerging chemical contribution to the washing ageing could be counteracted by, e.g., protective coatings.

Nonetheless, the effect of polymer surface enhancement as well as protective coatings on washing durability needs to be addressed in further investigation. This comes along with the issue of modified washing conditions such as elevated temperature or washing additives, such as copper corrosion inhibitors [50]. For the basic understanding of the metal–polymer interfaces, more investigation into the role of the bound water must be focussed, i.e., if the contribution of cellulose is physical by increasing the mechanical surface properties, chemical by taking part in the bonding process or different. There is also the question of in what way the water supports the delamination of the metal from the polymer.

## Figures and Tables

**Table 1:** Parameters of the experimental setup for the dynamic flex tester.

Construction Parameter	Value
roll length	55 mm
final roll diameter	16 mm (with wrapped fabric)
roll–roll centre distance	22 mm
abradant	Lyocell, plain woven
bending angle α	86°
diameter crank rotation	50 mm
spring stiffness	0.046 ± 0.002 N/mm
maximum spring force in exp. setup	3 N
distance centre of roll and centre of crank	120 mm vertical/115 mm horizontal
distance spring attachment—roll	250 mm
power (motor)	72 W
rotation speed	60 rpm

**Table 2:** Energy loss and maximum Baud rate at different electrical resistances of a conductive line.

Resistance of a Conductive Line (Ω)	Lost Energy in Textile (W)			Max. Baud Rate
	40 mA	80 mA	160 mA	
10	0.032	0.128	0.512	921,000
160	0.512	2.048	8.192	921,000
250	0.800	3.200	12.800	921,000
500	1.600	6.400	25.600	576,000
1250	4.000	16.000	64.000	256,000
2700	8.640	34.560	138.240	115,200

## Data Availability

The data that support the findings of this study are available from the corresponding author upon reasonable request.

## Supplementary Materials

The following supporting information can be downloaded at: https://www.mdpi.com/article/10.3390/ma16030912/s1, Figure S1. Photo of wet dynamic flex testing with electric motor drive, online resistance measurement and washing solution supply, Figure S2. Sketch of the specimen mounted to the new dynamic flex tester with the estimated assigned impact areas, Figure S3. Failure of substrate during cyclic abrasion., Figure S4. Measured elongation for fixed strain rates of 2.5 cm broad specimens of Lyocell and PA 66, cycling from 0 to 3.8 N/mm², 9.0 N/mm² and 15 N/mm², Figure S5. resistance dependent voltage input for the required electrical power; three data points are highlighted at a textile resistance of 200 Ω, 500 Ω and 1000 Ω, which would require 21 V, 45 V and 85 V at a current of 40 mA.
