# Supplementary material for: Investigation of the Functional Ageing of Conductive Coated Fabrics under Simulated Washing Conditions
Source: Materials (Basel). 2023 Jan 18;16(3):912. doi: 10.3390/ma16030912 (PMC9918278; doi:10.3390/ma16030912)
Supplement: Supplementary file 1 [file materials-16-00912-s001.zip › materials-2146455-supplementary.pdf]

Investigation of the Functional Ageing of Conductive Coated Fabrics under Simulated Washing Conditions

Christian Biermaier [1], Phillip Petz [2], Thomas Bechtold [1,*] and Tung Pham [1,*]

1 Universität Innsbruck, Research Institute of Textile Chemistry and Textile Physics, , Hoechsterstrasse 73, 6850 Dornbirn, Austria
2.Embedded Systems Lab, University of Applied Sciences Upper Austria, 4232 Hagenberg, Austria
* Correspondence: thomas.bechtold@uibk.ac.at (T.B.); tung.pham@uibk.ac.at (T.P.)

Supporting Information

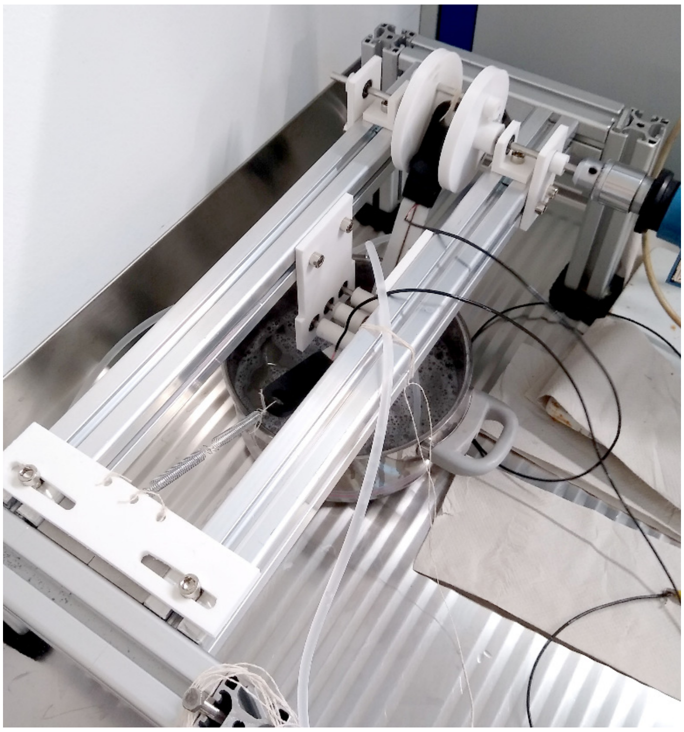

Figure S1. *Photo of wet dynamic flex testing with electric motor drive, online resistance measurement and washing solution supply.*

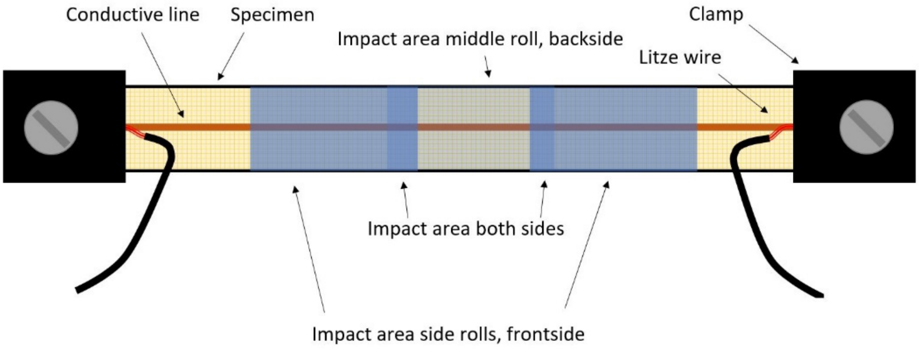

Figure S2. *Sketch of the specimen mounted to the new dynamic flex tester with the estimated assigned impact areas.*

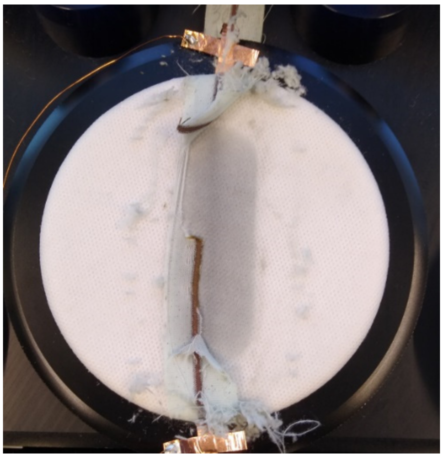

Figure S3. *Failure of substrate during cyclic abrasion.*

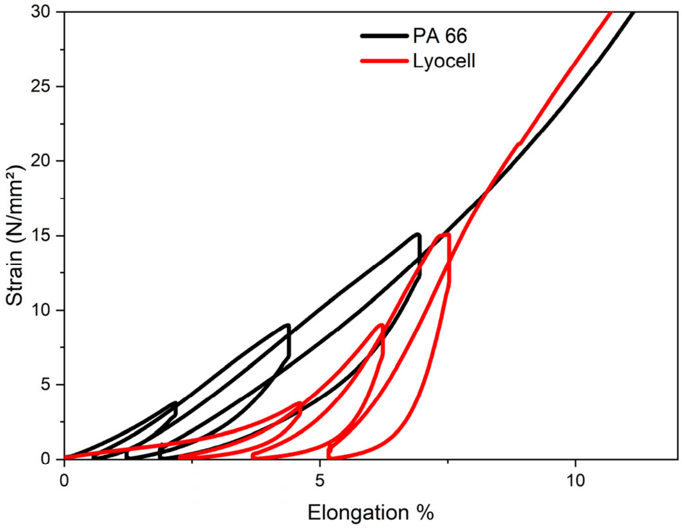

Figure S4. *Measured elongation for fixed strain rates of 2.5 cm broad specimens of Lyocell and PA 66, cycling from 0 to 3.8 N/mm², 9.0 N/mm² and 15 N/mm².*

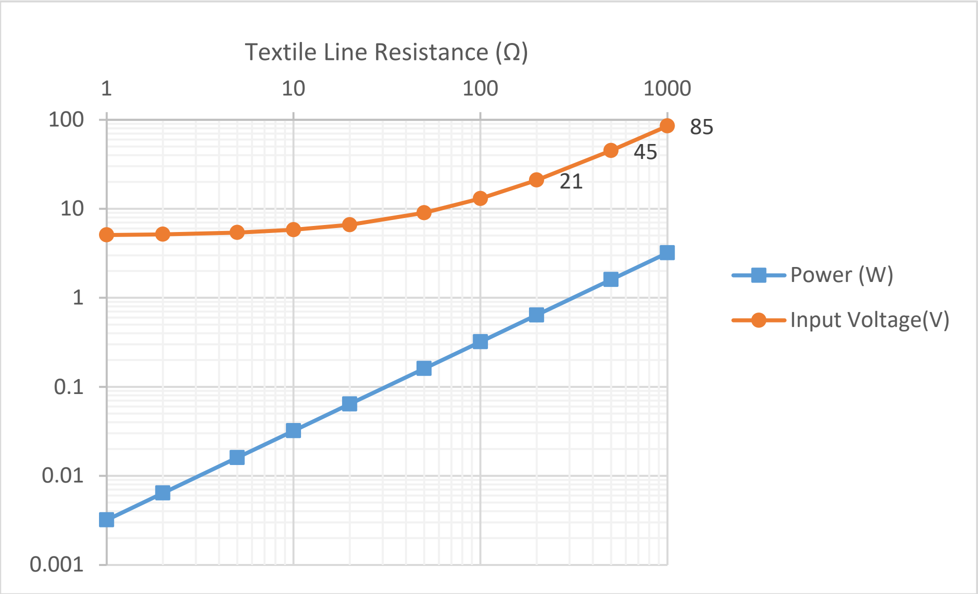

Figure S5. *resistance dependent voltage input for the required electrical power; three data points are highlighted at a textile resistance of 200 Ω, 500 Ω and 1000 Ω, which would require 21 V, 45 V and 85 V at a current of 40 mA.*
